# A pilot study to identify clinical predictors for wrist fractures in adult patients with acute wrist injury

**DOI:** 10.1186/s12245-015-0050-y

**Published:** 2015-02-11

**Authors:** Anne Brants, Michiel A IJsseldijk

**Affiliations:** Emergency Department, Canisius-Wilhelmina Ziekenhuis (CWZ), Postbox 9015, 6500 GS Nijmegen, the Netherlands; RadboudUMC, Comeniuslaan 4, 6525 HP Nijmegen, the Netherlands

**Keywords:** Acute wrist trauma, Wrist fracture, Clinical predictors

## Abstract

**Background:**

To date, no clinical decision rules for acute wrist injuries are available. In the past, clinical decision rules for the knee, ankle and spine injuries have been developed and validated. Implementation of these rules resulted in standardised clinical assessment at the emergency department and a substantial reduction of radiographic diagnostics. The objective of the study was to identify predictors for wrist fractures in patients with acute wrist injury which might potentiate a clinical decision rule in the future. This is a prospective pilot study in adult patients presenting with acute wrist injury at the emergency department of the Canisius-Wilhelmina Hospital in the Netherlands.

**Methods:**

Clinical variables were collected in a case report file by emergency physicians. Radiography was ordered according to common practice to confirm or rule out the presence of fractures. Independent associations between the presence of clinical variables and wrist fractures were calculated. Multivariable analysis was performed in order to quantify sensitivity and specificity for fracture prediction.

**Results:**

A total of 63 wrist fractures were detected in the study population of 95. Age over 55 years, inability to carry weight directly after trauma, support of injured wrist by the contralateral hand for pain relief, presence of swelling and/or hematoma, visible wrist deformity and reduced range of motion were associated with the presence of a wrist fracture.

**Conclusions:**

Our study identified clinical predictors for wrist fractures in patients with acute wrist injury. Future studies are needed for justification of evidence-based wrist assessment and identification of a 100% sensitive decision rule for wrist fractures.

## Background

Distal radius fractures are the most prevalent wrist fracture and are one of the most common fractures in both adults [1] and children [2,3]. The majority of patients with acute wrist injury visit the emergency department (ED), and although radiography is the gold standard in diagnosing wrist fractures [4,5], a detailed history alone may lead to a specific diagnosis in approximately 70% of patients with wrist pain [6].

Despite availability of validated clinical decision rules for knee [7], ankle [8,9] and cervical injuries [10], no validated clinical decision rules exist for wrist injuries, and studies in this field are scarce. Clinical features found to predict upper extremity fractures include the following: radial tenderness [11-15], gross deformity [13], difference in grip strength between the injured and non-injured arm [11], focal swelling [14,15], abnormal supination or pronation [14] and pain with motion [15]. However, those studies were non-specific for wrist fractures and only included paediatric patients [11-15].

The validated clinical decision rules for ankle, knee and cervical injuries have led to effective standardised clinical patient assessment, leading to effective standardised diagnostics and a decline in radiography as a diagnostic tool. Moreover, a substantial reduction in health-care costs was realised [16]. Radiologic assessment is used routinely as a diagnostic tool to rule out a wrist fracture due to the lack of clinical decision rules. Previous studies have shown that nearly half of all wrist radiographs do not show a fracture [17,18]. A clinical decision rule might aid in more effective use of radiography for wrist injuries. To accomplish development of a clinical decision rule in the future, quantification of the predictive value of clinical variables for wrist fractures is an essential first step.

The objective of this pilot study is to quantify the predictive value of clinical features for wrist fractures in an adult population and to combine the significant clinical variables in order to define a clinical decision rule with a 100% sensitivity for our population.

## Methods

### Study design and population

This prospective pilot study was conducted between February 2013 and August 2013 at the ED of the Canisius-Wilhelmina Hospital (CWZ), a top urban clinical teaching hospital in the Netherlands. All adult patients presenting with an acute wrist trauma to the ED at CWZ were eligible for this study.

The wrist anatomically includes the area formed by the distal 5 cm of the radius and ulna, the eight carpal bones and the overlying soft tissue. Acute wrist injury was defined as any blunt trauma to the wrist. Exclusion criteria were as follows: age below 18 years, referral by radiology, severe cognitive impairment resulting in a lack of effective communication, wrist injury sustained more than a week before presentation at the ED, reassessment of a wrist injury or solitary skin lesions.

Emergency physicians completed a case report form (CRF) for every patient with acute wrist injury. X-rays were ordered according to common practice. Patient assessment and registration was performed prior to radiographic assessment to ensure that emergency physicians were blinded to radiographic results.

### Data collection and measurement

All patients gave informed consent prior to the start of the wrist examination. Only fully qualified emergency physicians were involved in the assessment of wrist injuries at the ED. The clinical data recorded in the CRF was based on the available literature regarding examination of acute wrist injuries [19]. To ensure standardised data collection and registration, all emergency physicians were instructed how to perform clinical wrist examination and how to complete the CRF prior to the start of the study. Numerous variables were assessed: patient characteristics, history characteristics, clinical features with numerous points of tenderness and six ranges of motion.

All radiographs were assessed by the radiologist on call at the time of treatment. Radiographic outcomes were defined as fracture or no fracture. Although radiography is regarded as the standard of practice, a fracture can remain undetected [20]. To account for this, all radiographs were assessed by a second radiologist, and all medical files of the patients were checked to see if a fracture was diagnosed during the outpatient follow-up within 30 days after primary presentation at the ED. Interobserver reliability was calculated for radiographic outcomes.

### Outcome variables and data analysis

The primary outcome measure was presence or absence of a fracture, which was based on the conclusion of the radiologist. All clinical variables were assessed independently regarding their association with fractures by univariate analysis with the *χ*^2^-test. If possible, 95% confidence intervals (CI) were calculated. Patient age was dichotomised in cut-off value with the most discriminating power for a fracture. Only clinical variables that were significantly associated with a fracture (*ρ* < 0.10) were included in a multivariate model with the goal to develop a decision rule that was 100% sensitive for wrist fractures in this population with a specificity as highly as possible. Statistical Package for Social Statistics Version 21.0 (SPSS) was used to analyse data.

### Medical ethical committee

The local medical ethical committee (METC) approved the study design and provided a waiver of the requirement for informed consent due to the descriptive character of the study and absence of interventions. Nevertheless, informed consent was obtained from all patients. The study received no financial support. No competing interests were declared.

## Results

### Study population and characteristics

During the pilot, a total of 95 wrist injuries were included. Ten physicians were involved in the clinical wrist assessment, and the variables were assessed and registered in a standardised manner. Radiographic assessment was ordered according to common practice. Table 1 shows the characteristics of the study population. In case of bilateral wrist injury, both wrists were regarded as separate cases. The mean age of the study population was 50 years old, and 64% of the patients were female. The most common trauma mechanism was a fall on an outstretched hand. The emergency physician ordered an X-ray in all cases. A total of 63 fractures were diagnosed, a fracture rate of 66%. The most commonly diagnosed fracture is that of the distal radius. In the majority of the cases, patients received treatment with cast immobilisation, while fracture reduction was performed in 15% of the study population and surgery in the operating room was required in 4% of the cases.

**Table 1:** **Study characteristics (** ***N*** **= 95)**

**Patient characteristics**	**Value**
Age	
Mean in years (sd)	51.45 (20.27)
Median in years (range)	50.00 (18 to 97)
Gender, *n* (%)	
Male	30 (32)
Female	64 (67)
Unknown	1 (1)
Injured site, *n* (%)	
Right	47 (50)
Left	48 (51)
Action leading to injury, *n* (%)	
Fall from height	9 (10)
Traffic accident	14 (15)
Simple fall	55 (58)
Sports	7 (7)
Others	9 (10)
Unknown	1 (1)
Trauma mechanism, *n* (%)	
Fall on outstretched hand	42 (44)
Hyperextension	5 (5)
Direct force	6 (6)
Others	8 (8)
Unknown	30 (28)
Diagnosis, *n* (%)	
Fracture	63 (66)
Distal radial fracture	43 (45)
Distal ulnar fracture	7 (7)
Scaphoid fracture	9 (9)
Carpal fracture (excluding scaphoid)	3 (3)
Other fractures	1 (1)
Soft tissue injury	32 (34)
Treatment, *n* (%)	
None	12 (13)
Pressure tape	6 (6)
Immobilisation	52 (55)
Reponation	14 (15)
Surgery	4 (4)
Others	7 (7)

### Radiographic outcome and identification of clinical predictors for wrist fractures

Presence of a wrist fracture was based on the radiological outcome. One additional fracture was diagnosed by the second radiographic observer. Interobserver variability for the presence of a fracture based on radiology was good with a kappa of 0.70 (0.071) and *ρ* < 0.000. Univariate analysis was performed to see whether clinical features were independently associated with the presence of a wrist fracture. Table 2 shows the results of univariate analysis. Significant predictors for wrist fractures were as follows: age of 55 or above, inability to carry weight directly after trauma, support of injured wrist by contralateral hand for pain relief and swelling of wrist directly after trauma. Significant variables derived from wrist assessment were as follows: visible wrist deformity, presence of swelling and/or hematoma, reduced radial deviation, reduced palmar flexion and reduced supination.

**Table 2:** **The association between clinical variables and presence of a fracture (** ***N*** **= 95)**

**Clinical variable**	***χ*** ^**2**^ **-test**	***Ρ*** **value**
Patient history		
Age >55 years	6.660	0.0036
Setting trauma high-low risk	1.038	0.308
‘Cracking’ sound heard	2.124	0.36
Inability to continue activity after trauma	0.69	0.723
Inability to carry weight directly after trauma	5.219	0.074
Support injured wrist by contralateral hand for pain relief	9.929	0.002
Swelling of wrist directly after trauma	16.213	0.000
Reduced range of motion directly after trauma	2.307	0.316
Wrist assessment		
Swelling and/or hematoma present	14.703	0.000
Visible wrist deformity	6.976	0.0088
Tenderness at wrist^a^	-	-
Pain distal radius with axial compression	0.856	0.652
Painful compression DRUJ^b^	1.50	0.463
Reduced range of motion^c^	9.158	0.002
Reduced radial deviation	5.095	0.024
Reduced ulnar deviation	1.776	0.183
Reduced palmar flexion	10.815	0.001
Reduced dorsoflexion	1.702	0.000
Reduced pronation	2.304	0.129
Reduced supination	9.460	0.002

^a^The *χ* ^2^-test and *Ρ* value were impossible to calculate since all patients had wrist tenderness. ^b^Distal radio-ulnar joint. ^c^The variable reduced range of motion was used in the decision model rather than separated reduced ranges of motion.

### The construction of a clinical decision rule for this pilot population

All clinical features with *ρ* < 0.10 were entered into a logistics regression model. Different sets of decision rules were tried in order to develop one decision rule with 100% sensitivity for fractures. Decision rules with a higher specificity at the cost of missing fractures were considered unsuitable. The final decision rule for our population was based on the following elements: 1) age ≥55 years, 2) inability to continue activity after trauma, 3) support injured wrist by contralateral hand for pain relief, 4) wrist deformity, 5) swelling and/or hematoma and 5) reduced range of wrist motion. Table 3 shows the performance of the target clinical decision rule in our study population. It identified all fractures; however, it also identified 16 patients without a fracture incorrectly.

**Table 3:** **Performance of the clinical decision rule (** ***N*** **= 95)**

	**Fracture present**	**Fracture absent**
Fracture predicted		
Yes	63	16
No	0	16
Sensitivity	1.00 (95% CI 0.93 to 1.00)	
Specificity	0.50 (95% CI 0.32 to 0.68)	
Positive predictive value	0.80 (95% CI 0.69 to 0.88)	
Negative predictive value	1.00 (95% CI 0.76 to 1.00)	
Patients correctly identified	83.2%	
Radiographic rate reduction	100% to 83.2% = 16.8%	

The positive predictive value for a wrist fracture was 0.80 (95%CI 0.69 to 0.88), and the negative predictive value for a wrist fracture was 1.00 (95%CI 0.76 to 1.00). The clinical decision rule had a sensitivity of 1.00 (95%CI 0.93 to 1.00) and specificity of 0.50 (95%CI 0.32 to 0.68). Application of this clinical decision rule would have led to a radiography rate of 83%, which means a reduction of 17% without missing any wrist fractures.

## Discussion

This pilot study aimed to identify predictors for wrist fractures and covered the full spectrum of adult patients with acute wrist injury. The study sample was heterogeneous regarding trauma mechanisms and had a wide range in patient age. Thompson [21], who studied the epidemiology of distal radius fractures, found that the incidence of wrist fractures had a female:male ratio of 3.9:1. In our cohort, two thirds of the patients were female. No significant association between the female gender and presence of a fracture was found (*P* = 0.404). This might be attributed to the small sample size of our cohort.

Our purpose was to identify variables from both patient history as wrist assessment with a predictive value for wrist fractures. Emergency physicians are only likely to support a clinical decision rule if it is based on clinical variables that are easy and not time consuming to check [22]. In our cohort, a number of variables were found to be independently associated with the presence of a fracture. Our results share both differences as similarities with previous studies.

Age of 55 or above and the inability to carry weight directly after trauma were independently associated with the presence of a wrist fracture in our cohort. We also tried the cut-off values of 50 and 60 years, but 55 years had the highest predictive value. This result is in line with what has been described in the literature [8]. The incidence of fractures increases with age and shows an increase after the menopause due to osteoporosis [21].

To our knowledge, the inability to carry weight as a predictor for wrist fractures was never tested before. We adopted this variable from the Ottowa ankle rules, and it seems that its predictive power may be applicable to wrist injuries as well.

However, the majority of the significant predictors for wrist fractures in our cohort was based on clinical wrist assessment. Multiple studies [11-15,23] found associations between visible wrist deformity, presence of hematoma or swelling and reduced range of motion and the presence of a fracture. Except for localised tenderness, we identified the same predictors, and therefore, associations between wrist deformity, presence of hematoma or swelling, reduced range of motion and wrist fractures seem justified. Localised tenderness [11-15] did not discriminate between a fracture or contusion in our population because it was present in all patients. We believe that radial tenderness is the main reason for patients to present themselves at the emergency department irrespective of the presence of a possible fracture or soft tissue injury, and therefore, it is not a disciminating factor. Logically, wrist deformity was found to be a strong predictor of a fracture in our cohort. We agree with Cevik [23] that a wrist deformity should be treated as a fracture until proven otherwise and forms a strong indication for wrist radiography.

Abnormal supination or pronation [14] and pain with motion [15] have been associated with fractures in past studies. Four of the six tested ranges of motion were associated with the presence of a fracture. Reduced ulnar deviation and reduced pronation were not, most likely due to the heterogeneity of the fracture locations and therefore different symptomatology. We believe that any reduced range of wrist motion is an indication for radiographic assessment.

We found a radiography rate of 100% in our study, which shows that emergency physicians appear reluctant to rule out a fracture based on clinical judgement alone. In a recent study by van den Brand [18], a radiography rate of 91.5% was found while missing at least four fractures. In times of evidence-based medicine and pressure on health-care costs, it is essential to balance the risk of missing a fracture with the costs of radiographic assessment and exposure to radiation. We agree with van den Brand that it will be difficult to define a decision rule due to the high fracture ratio in wrists and might therefore not reach the same relative efficiency as the decision rules for knee and ankle trauma [7,8]. However the application of the decision rule for wrist injury in our population would have led in a radiographic reduction of 17% without missing any fractures. Therefore, a clinical decision rule for wrist injuries could lead to a less conservative approach to wrist injuries and a small reduction in radiography. This view is supported by our colleagues in Amsterdam [7], who recently started a large multicentre study to identify a decision rule for wrist trauma.

We composed a decision rule based on the presence of any of the following variables: age ≥55 years, inability to continue activity after trauma, support injured wrist by contralateral hand for pain relief, wrist deformity, swelling/hematoma and reduced range of motion. However, our rule lacks prospective validation, and therefore, generalisation of our results is premature. In the future, large cohort studies like the multicentre study of the Amsterdam Wrist Rules Study Group [24] are needed to establish evidence-based wrist assessment.

## Conclusions

The clinical predictors for wrist fractures identified in this population are in line with what is found in previous studies. Future studies are needed for evidence-based wrist assessment and validation of a decision rule for acute wrist injury.

## Abbreviations

CRF: case report form
CI: confidence interval
CWZ: Canisius-Wilhelmina Ziekenhuis
DRUJ: distal radio-ulnar joint
ED: emergency department
OR: odds ratio
RadboudUMC: University Medical Centre (RadboudUMC)
SPSS: Statistical Package for Social Statistics Version 21.0
